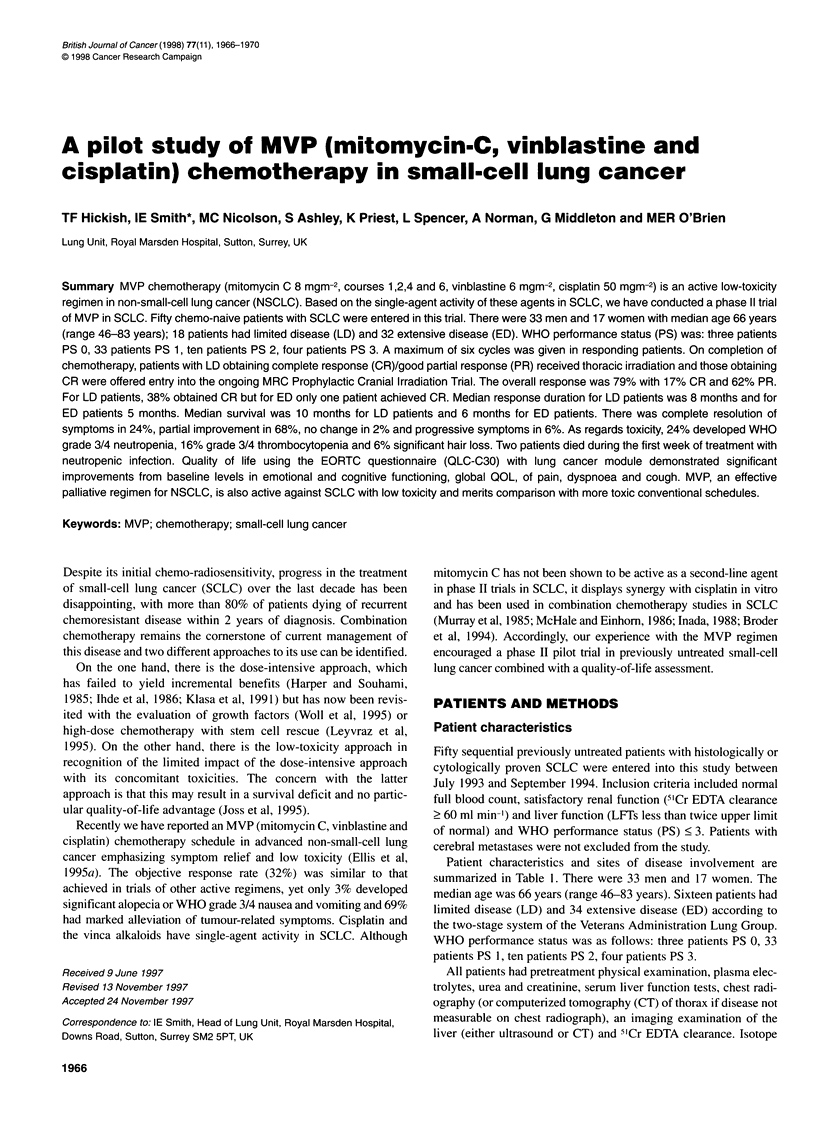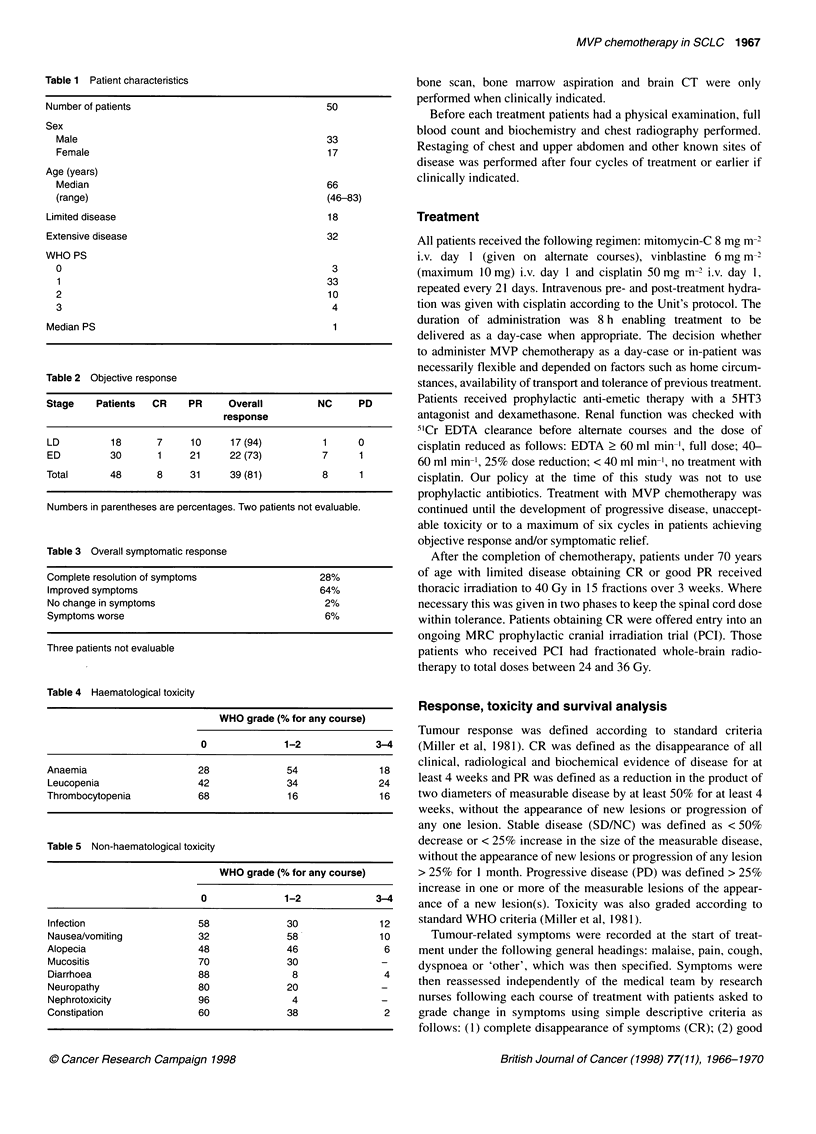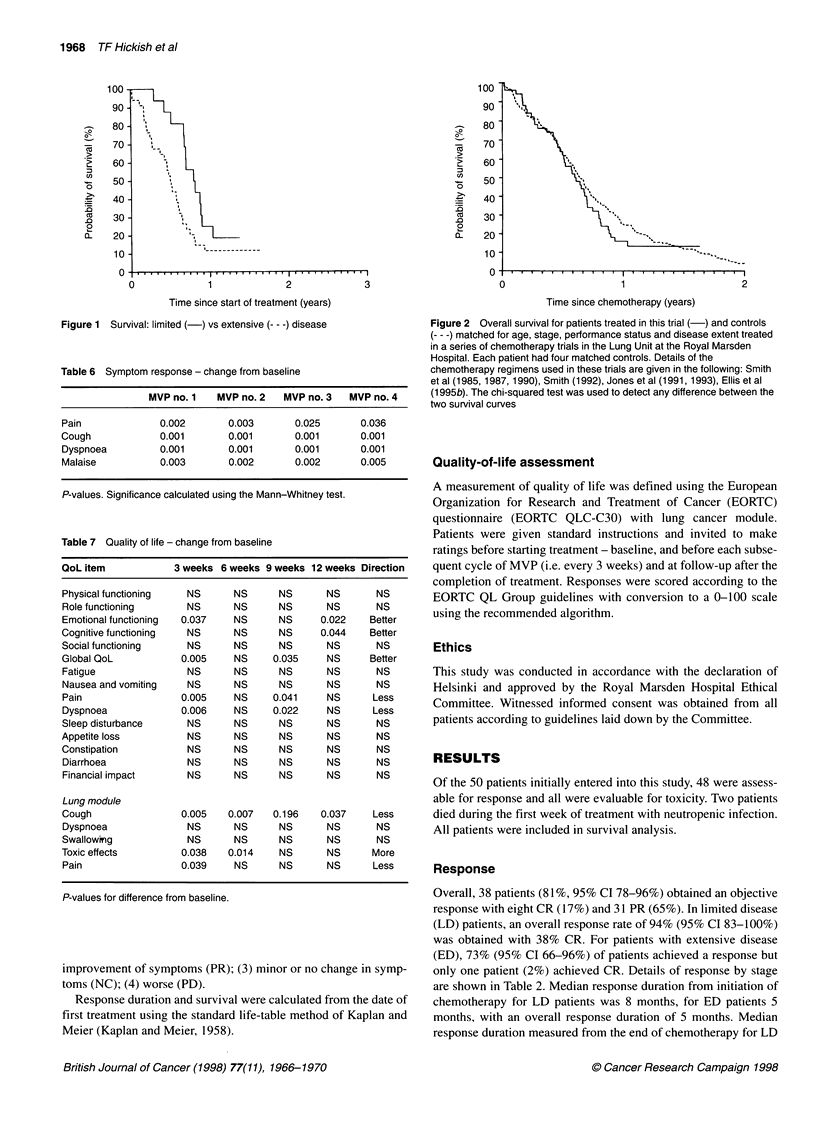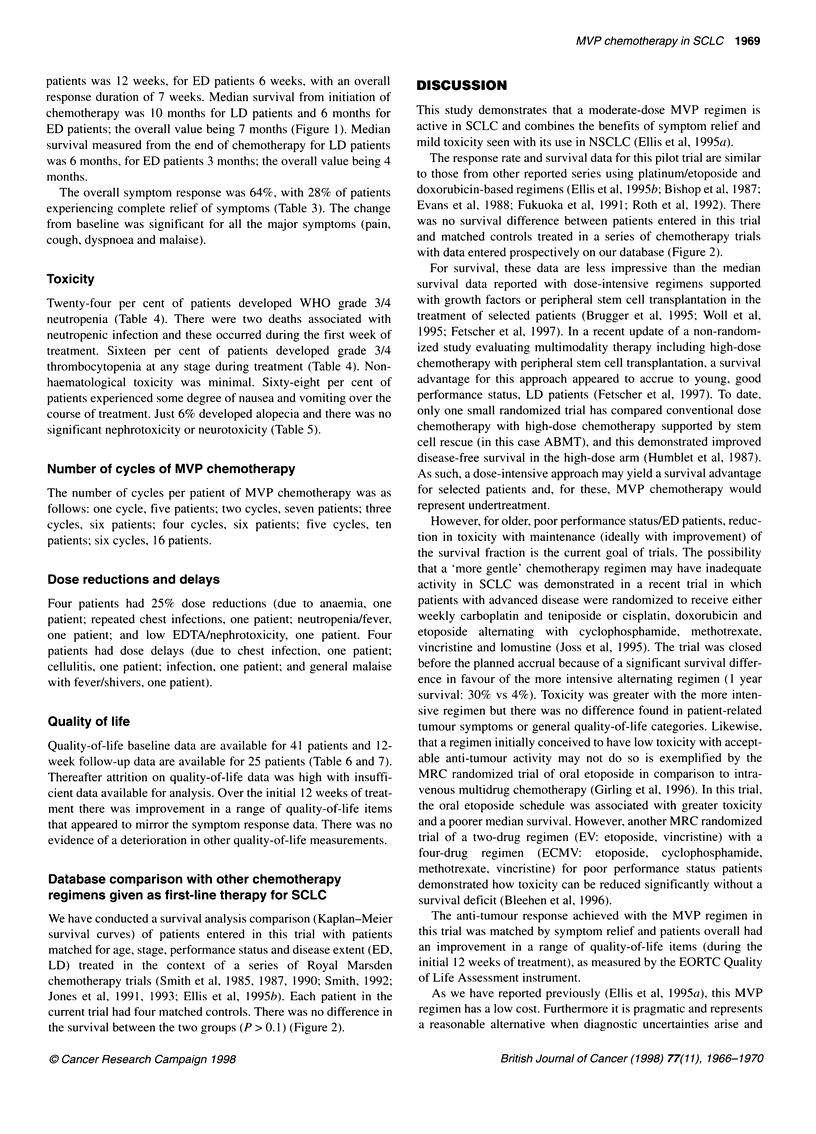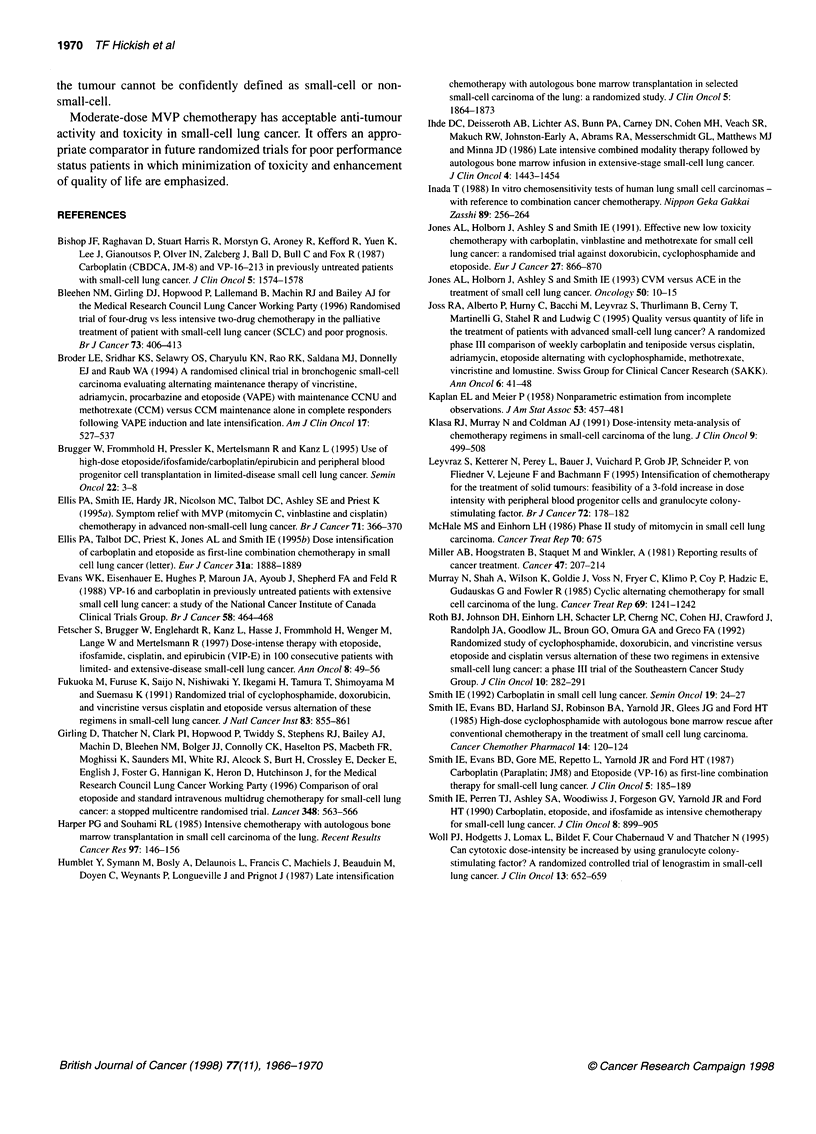# A pilot study of MVP (mitomycin-C, vinblastine and cisplatin) chemotherapy in small-cell lung cancer.

**DOI:** 10.1038/bjc.1998.326

**Published:** 1998-06

**Authors:** T. F. Hickish, I. E. Smith, M. C. Nicolson, S. Ashley, K. Priest, L. Spencer, A. Norman, G. Middleton, M. E. O'Brien

**Affiliations:** Lung Unit, Royal Marsden Hospital, Sutton, Surrey, UK.

## Abstract

MVP chemotherapy (mitomycin C 8 mg m(-2), courses 1, 2, 4 and 6, vinblastine 6 mg m(-2), cisplatin 50 mg m(-2)) is an active low-toxicity regimen in non-small-cell lung cancer (NSCLC). Based on the single-agent activity of these agents in SCLC, we have conducted a phase II trial of MVP in SCLC. Fifty chemo-naive patients with SCLC were entered in this trial. There were 33 men and 17 women with median age 66 years (range 46-83 years); 18 patients had limited disease (LD) and 32 extensive disease (ED). WHO performance status (PS) was: three patients PS 0, 33 patients PS 1, ten patients PS 2, four patients PS 3. A maximum of six cycles was given in responding patients. On completion of chemotherapy, patients with LD obtaining complete response (CR)/good partial response (PR) received thoracic irradiation and those obtaining CR were offered entry into the ongoing MRC Prophylactic Cranial Irradiation Trial. The overall response was 79% with 17% CR and 62% PR. For LD patients, 38% obtained CR but for ED only one patient achieved CR. Median response duration for LD patients was 8 months and for ED patients 5 months. Median survival was 10 months for LD patients and 6 months for ED patients. There was complete resolution of symptoms in 24%, partial improvement in 68%, no change in 2% and progressive symptoms in 6%. As regards toxicity, 24% developed WHO grade 3/4 neutropenia, 16% grade 3/4 thrombocytopenia and 6% significant hair loss. Two patients died during the first week of treatment with neutropenic infection. Quality of life using the EORTC questionnaire (QLC-C30) with lung cancer module demonstrated significant improvements from baseline levels in emotional and cognitive functioning, global QOL, of pain, dyspnoea and cough. MVP, an effective palliative regimen for NSCLC, is also active against SCLC with low toxicity and merits comparison with more toxic conventional schedules.


British Journal of Cancer (1998) 77(11), 1966–1970
© 1998 Cancer Research Campaign

### A pilot study of MVP (mitomycin-C, vinblastine and cisplatin) chemotherapy in small-cell lung cancer

**TF Hickish, IE Smith*, MC Nicolson, S Ashley, K Priest, L Spencer, A Norman, G Middleton and MER O'Brien**

Lung Unit, Royal Marsden Hospital, Sutton, Surrey, UK

**Summary** MVP chemotherapy (mitomycin C 8 mgm$^{-2}$, courses 1,2,4 and 6, vinblastine 6 mgm$^{-2}$, cisplatin 50 mgm$^{-2}$) is an active low-toxicity regimen in non-small-cell lung cancer (NSCLC). Based on the single-agent activity of these agents in SCLC, we have conducted a phase II trial of MVP in SCLC. Fifty chemo-naive patients with SCLC were entered in this trial. There were 33 men and 17 women with median age 66 years (range 46–83 years); 18 patients had limited disease (LD) and 32 extensive disease (ED). WHO performance status (PS) was: three patients PS 0, 33 patients PS 1, ten patients PS 2, four patients PS 3. A maximum of six cycles was given in responding patients. On completion of chemotherapy, patients with LD obtaining complete response (CR)/good partial response (PR) received thoracic irradiation and those obtaining CR were offered entry into the ongoing MRC Prophylactic Cranial Irradiation Trial. The overall response was 79% with 17% CR and 62% PR. For LD patients, 38% obtained CR but for ED only one patient achieved CR. Median response duration for LD patients was 8 months and for ED patients 5 months. Median survival was 10 months for LD patients and 6 months for ED patients. There was complete resolution of symptoms in 24%, partial improvement in 68%, no change in 2% and progressive symptoms in 6%. As regards toxicity, 24% developed WHO grade 3/4 neutropenia, 16% grade 3/4 thrombocytopenia and 6% significant hair loss. Two patients died during the first week of treatment with neutropenic infection. Quality of life using the EORTC questionnaire (QLC-C30) with lung cancer module demonstrated significant improvements from baseline levels in emotional and cognitive functioning, global QOL, of pain, dyspnoea and cough. MVP, an effective palliative regimen for NSCLC, is also active against SCLC with low toxicity and merits comparison with more toxic conventional schedules.

**Keywords:** MVP; chemotherapy; small-cell lung cancer

Despite its initial chemo-radiosensitivity, progress in the treatment of small-cell lung cancer (SCLC) over the last decade has been disappointing, with more than 80% of patients dying of recurrent chemoresistant disease within 2 years of diagnosis. Combination chemotherapy remains the cornerstone of current management of this disease and two different approaches to its use can be identified.

On the one hand, there is the dose-intensive approach, which has failed to yield incremental benefits (Harper and Souhami, 1985; Ihde et al, 1986; Klasa et al, 1991) but has now been revisited with the evaluation of growth factors (Woll et al, 1995) or high-dose chemotherapy with stem cell rescue (Leyvraz et al, 1995). On the other hand, there is the low-toxicity approach in recognition of the limited impact of the dose-intensive approach with its concomitant toxicities. The concern with the latter approach is that this may result in a survival deficit and no particular quality-of-life advantage (Joss et al, 1995).

Recently we have reported an MVP (mitomycin C, vinblastine and cisplatin) chemotherapy schedule in advanced non-small-cell lung cancer emphasizing symptom relief and low toxicity (Ellis et al, 1995*a*). The objective response rate (32%) was similar to that achieved in trials of other active regimens, yet only 3% developed significant alopecia or WHO grade 3/4 nausea and vomiting and 69% had marked alleviation of tumour-related symptoms. Cisplatin and the vinca alkaloids have single-agent activity in SCLC. Although mitomycin C has not been shown to be active as a second-line agent in phase II trials in SCLC, it displays synergy with cisplatin in vitro and has been used in combination chemotherapy studies in SCLC (Murray et al, 1985; McHale and Einhorn, 1986; Inada, 1988; Broder et al, 1994). Accordingly, our experience with the MVP regimen encouraged a phase II pilot trial in previously untreated small-cell lung cancer combined with a quality-of-life assessment.

*Received 9 June 1997*
*Revised 13 November 1997*
*Accepted 24 November 1997*

*Correspondence to:* IE Smith, Head of Lung Unit, Royal Marsden Hospital, Downs Road, Sutton, Surrey SM2 5PT, UK

### PATIENTS AND METHODS

### Patient characteristics

Fifty sequential previously untreated patients with histologically or cytologically proven SCLC were entered into this study between July 1993 and September 1994. Inclusion criteria included normal full blood count, satisfactory renal function ($^{51}$Cr EDTA clearance $\geq$ 60 ml min$^{-1}$) and liver function (LFTs less than twice upper limit of normal) and WHO performance status (PS) $\leq$ 3. Patients with cerebral metastases were not excluded from the study.

Patient characteristics and sites of disease involvement are summarized in Table 1. There were 33 men and 17 women. The median age was 66 years (range 46–83 years). Sixteen patients had limited disease (LD) and 34 extensive disease (ED) according to the two-stage system of the Veterans Administration Lung Group. WHO performance status was as follows: three patients PS 0, 33 patients PS 1, ten patients PS 2, four patients PS 3.

All patients had pretreatment physical examination, plasma electrolytes, urea and creatinine, serum liver function tests, chest radiography (or computerized tomography (CT) of thorax if disease not measurable on chest radiograph), an imaging examination of the liver (either ultrasound or CT) and $^{51}$Cr EDTA clearance. Isotope

**Table 1** Patient characteristics

| | |
|---|---|
| Number of patients | 50 |
| Sex | |
| Male | 33 |
| Female | 17 |
| Age (years) | |
| Median | 66 |
| (range) | (46–83) |
| Limited disease | 18 |
| Extensive disease | 32 |
| WHO PS | |
| 0 | 3 |
| 1 | 33 |
| 2 | 10 |
| 3 | 4 |
| Median PS | 1 |

**Table 2** Objective response

| Stage | Patients | CR | PR | Overall response | NC | PD |
|---|---|---|---|---|---|---|
| LD | 18 | 7 | 10 | 17 (94) | 1 | 0 |
| ED | 30 | 1 | 21 | 22 (73) | 7 | 1 |
| Total | 48 | 8 | 31 | 39 (81) | 8 | 1 |

Numbers in parentheses are percentages. Two patients not evaluable.

**Table 3** Overall symptomatic response

| | |
|---|---|
| Complete resolution of symptoms | 28% |
| Improved symptoms | 64% |
| No change in symptoms | 2% |
| Symptoms worse | 6% |

Three patients not evaluable

**Table 4** Haematological toxicity

| | WHO grade (% for any course) | | |
|---|---|---|---|
| | 0 | 1–2 | 3–4 |
| Anaemia | 28 | 54 | 18 |
| Leucopenia | 42 | 34 | 24 |
| Thrombocytopenia | 68 | 16 | 16 |

**Table 5** Non-haematological toxicity

| | WHO grade (% for any course) | | |
|---|---|---|---|
| | 0 | 1–2 | 3–4 |
| Infection | 58 | 30 | 12 |
| Nausea/vomiting | 32 | 58 | 10 |
| Alopecia | 48 | 46 | 6 |
| Mucositis | 70 | 30 | – |
| Diarrhoea | 88 | 8 | 4 |
| Neuropathy | 80 | 20 | – |
| Nephrotoxicity | 96 | 4 | – |
| Constipation | 60 | 38 | 2 |

bone scan, bone marrow aspiration and brain CT were only performed when clinically indicated.

Before each treatment patients had a physical examination, full blood count and biochemistry and chest radiography performed. Restaging of chest and upper abdomen and other known sites of disease was performed after four cycles of treatment or earlier if clinically indicated.

### Treatment

All patients received the following regimen: mitomycin-C 8 mg $m^{-2}$ i.v. day 1 (given on alternate courses), vinblastine 6 mg $m^{-2}$ (maximum 10 mg) i.v. day 1 and cisplatin 50 mg $m^{-2}$ i.v. day 1, repeated every 21 days. Intravenous pre- and post-treatment hydration was given with cisplatin according to the Unit's protocol. The duration of administration was 8 h enabling treatment to be delivered as a day-case when appropriate. The decision whether to administer MVP chemotherapy as a day-case or in-patient was necessarily flexible and depended on factors such as home circumstances, availability of transport and tolerance of previous treatment. Patients received prophylactic anti-emetic therapy with a 5HT3 antagonist and dexamethasone. Renal function was checked with $^{51}$Cr EDTA clearance before alternate courses and the dose of cisplatin reduced as follows: EDTA $\geq$ 60 ml $min^{-1}$, full dose; 40–60 ml $min^{-1}$, 25% dose reduction; < 40 ml $min^{-1}$, no treatment with cisplatin. Our policy at the time of this study was not to use prophylactic antibiotics. Treatment with MVP chemotherapy was continued until the development of progressive disease, unacceptable toxicity or to a maximum of six cycles in patients achieving objective response and/or symptomatic relief.

After the completion of chemotherapy, patients under 70 years of age with limited disease obtaining CR or good PR received thoracic irradiation to 40 Gy in 15 fractions over 3 weeks. Where necessary this was given in two phases to keep the spinal cord dose within tolerance. Patients obtaining CR were offered entry into an ongoing MRC prophylactic cranial irradiation trial (PCI). Those patients who received PCI had fractionated whole-brain radiotherapy to total doses between 24 and 36 Gy.

### Response, toxicity and survival analysis

Tumour response was defined according to standard criteria (Miller et al, 1981). CR was defined as the disappearance of all clinical, radiological and biochemical evidence of disease for at least 4 weeks and PR was defined as a reduction in the product of two diameters of measurable disease by at least 50% for at least 4 weeks, without the appearance of new lesions or progression of any one lesion. Stable disease (SD/NC) was defined as < 50% decrease or < 25% increase in the size of the measurable disease, without the appearance of new lesions or progression of any lesion > 25% for 1 month. Progressive disease (PD) was defined > 25% increase in one or more of the measurable lesions of the appearance of a new lesion(s). Toxicity was also graded according to standard WHO criteria (Miller et al, 1981).

Tumour-related symptoms were recorded at the start of treatment under the following general headings: malaise, pain, cough, dyspnoea or 'other', which was then specified. Symptoms were then reassessed independently of the medical team by research nurses following each course of treatment with patients asked to grade change in symptoms using simple descriptive criteria as follows: (1) complete disappearance of symptoms (CR); (2) good

© Cancer Research Campaign 1998

Figure 1 Survival: limited (—) vs extensive (- - -) disease

Table 6 Symptom response – change from baseline

| | MVP no. 1 | MVP no. 2 | MVP no. 3 | MVP no. 4 |
|---|---|---|---|---|
| Pain | 0.002 | 0.003 | 0.025 | 0.036 |
| Cough | 0.001 | 0.001 | 0.001 | 0.001 |
| Dyspnoea | 0.001 | 0.001 | 0.001 | 0.001 |
| Malaise | 0.003 | 0.002 | 0.002 | 0.005 |

*P*-values. Significance calculated using the Mann–Whitney test.

Table 7 Quality of life – change from baseline

| QoL item | 3 weeks | 6 weeks | 9 weeks | 12 weeks | Direction |
|---|---|---|---|---|---|
| Physical functioning | NS | NS | NS | NS | NS |
| Role functioning | NS | NS | NS | NS | NS |
| Emotional functioning | 0.037 | NS | NS | 0.022 | Better |
| Cognitive functioning | NS | NS | NS | 0.044 | Better |
| Social functioning | NS | NS | NS | NS | NS |
| Global QoL | 0.005 | NS | 0.035 | NS | Better |
| Fatigue | NS | NS | NS | NS | NS |
| Nausea and vomiting | NS | NS | NS | NS | NS |
| Pain | 0.005 | NS | 0.041 | NS | Less |
| Dyspnoea | 0.006 | NS | 0.022 | NS | Less |
| Sleep disturbance | NS | NS | NS | NS | NS |
| Appetite loss | NS | NS | NS | NS | NS |
| Constipation | NS | NS | NS | NS | NS |
| Diarrhoea | NS | NS | NS | NS | NS |
| Financial impact | NS | NS | NS | NS | NS |
| *Lung module* | | | | | |
| Cough | 0.005 | 0.007 | 0.196 | 0.037 | Less |
| Dyspnoea | NS | NS | NS | NS | NS |
| Swallowing | NS | NS | NS | NS | NS |
| Toxic effects | 0.038 | 0.014 | NS | NS | More |
| Pain | 0.039 | NS | NS | NS | Less |

*P*-values for difference from baseline.

improvement of symptoms (PR); (3) minor or no change in symptoms (NC); (4) worse (PD).

Response duration and survival were calculated from the date of first treatment using the standard life-table method of Kaplan and Meier (Kaplan and Meier, 1958).

Figure 2 Overall survival for patients treated in this trial (—) and controls (- - -) matched for age, stage, performance status and disease extent treated in a series of chemotherapy trials in the Lung Unit at the Royal Marsden Hospital. Each patient had four matched controls. Details of the chemotherapy regimens used in these trials are given in the following: Smith et al (1985, 1987, 1990), Smith (1992), Jones et al (1991, 1993), Ellis et al (1995*b*). The chi-squared test was used to detect any difference between the two survival curves

### Quality-of-life assessment

A measurement of quality of life was defined using the European Organization for Research and Treatment of Cancer (EORTC) questionnaire (EORTC QLC-C30) with lung cancer module. Patients were given standard instructions and invited to make ratings before starting treatment – baseline, and before each subsequent cycle of MVP (i.e. every 3 weeks) and at follow-up after the completion of treatment. Responses were scored according to the EORTC QL Group guidelines with conversion to a 0–100 scale using the recommended algorithm.

### Ethics

This study was conducted in accordance with the declaration of Helsinki and approved by the Royal Marsden Hospital Ethical Committee. Witnessed informed consent was obtained from all patients according to guidelines laid down by the Committee.

### RESULTS

Of the 50 patients initially entered into this study, 48 were assessable for response and all were evaluable for toxicity. Two patients died during the first week of treatment with neutropenic infection. All patients were included in survival analysis.

### Response

Overall, 38 patients (81%, 95% CI 78–96%) obtained an objective response with eight CR (17%) and 31 PR (65%). In limited disease (LD) patients, an overall response rate of 94% (95% CI 83–100%) was obtained with 38% CR. For patients with extensive disease (ED), 73% (95% CI 66–96%) of patients achieved a response but only one patient (2%) achieved CR. Details of response by stage are shown in Table 2. Median response duration from initiation of chemotherapy for LD patients was 8 months, for ED patients 5 months, with an overall response duration of 5 months. Median response duration measured from the end of chemotherapy for LD

© Cancer Research Campaign 1998

patients was 12 weeks, for ED patients 6 weeks, with an overall response duration of 7 weeks. Median survival from initiation of chemotherapy was 10 months for LD patients and 6 months for ED patients; the overall value being 7 months (Figure 1). Median survival measured from the end of chemotherapy for LD patients was 6 months, for ED patients 3 months; the overall value being 4 months.

The overall symptom response was 64%, with 28% of patients experiencing complete relief of symptoms (Table 3). The change from baseline was significant for all the major symptoms (pain, cough, dyspnoea and malaise).

### Toxicity

Twenty-four per cent of patients developed WHO grade 3/4 neutropenia (Table 4). There were two deaths associated with neutropenic infection and these occurred during the first week of treatment. Sixteen per cent of patients developed grade 3/4 thrombocytopenia at any stage during treatment (Table 4). Non-haematological toxicity was minimal. Sixty-eight per cent of patients experienced some degree of nausea and vomiting over the course of treatment. Just 6% developed alopecia and there was no significant nephrotoxicity or neurotoxicity (Table 5).

### Number of cycles of MVP chemotherapy

The number of cycles per patient of MVP chemotherapy was as follows: one cycle, five patients; two cycles, seven patients; three cycles, six patients; four cycles, six patients; five cycles, ten patients; six cycles, 16 patients.

### Dose reductions and delays

Four patients had 25% dose reductions (due to anaemia, one patient; repeated chest infections, one patient; neutropenia/fever, one patient; and low EDTA/nephrotoxicity, one patient. Four patients had dose delays (due to chest infection, one patient; cellulitis, one patient; infection, one patient; and general malaise with fever/shivers, one patient).

### Quality of life

Quality-of-life baseline data are available for 41 patients and 12-week follow-up data are available for 25 patients (Table 6 and 7). Thereafter attrition on quality-of-life data was high with insufficient data available for analysis. Over the initial 12 weeks of treatment there was improvement in a range of quality-of-life items that appeared to mirror the symptom response data. There was no evidence of a deterioration in other quality-of-life measurements.

### Database comparison with other chemotherapy regimens given as first-line therapy for SCLC

We have conducted a survival analysis comparison (Kaplan–Meier survival curves) of patients entered in this trial with patients matched for age, stage, performance status and disease extent (ED, LD) treated in the context of a series of Royal Marsden chemotherapy trials (Smith et al, 1985, 1987, 1990; Smith, 1992; Jones et al, 1991, 1993; Ellis et al, 1995*b*). Each patient in the current trial had four matched controls. There was no difference in the survival between the two groups ($P > 0.1$) (Figure 2).

### DISCUSSION

This study demonstrates that a moderate-dose MVP regimen is active in SCLC and combines the benefits of symptom relief and mild toxicity seen with its use in NSCLC (Ellis et al, 1995*a*).

The response rate and survival data for this pilot trial are similar to those from other reported series using platinum/etoposide and doxorubicin-based regimens (Ellis et al, 1995*b*; Bishop et al, 1987; Evans et al, 1988; Fukuoka et al, 1991; Roth et al, 1992). There was no survival difference between patients entered in this trial and matched controls treated in a series of chemotherapy trials with data entered prospectively on our database (Figure 2).

For survival, these data are less impressive than the median survival data reported with dose-intensive regimens supported with growth factors or peripheral stem cell transplantation in the treatment of selected patients (Brugger et al, 1995; Woll et al, 1995; Fetscher et al, 1997). In a recent update of a non-randomized study evaluating multimodality therapy including high-dose chemotherapy with peripheral stem cell transplantation, a survival advantage for this approach appeared to accrue to young, good performance status, LD patients (Fetscher et al, 1997). To date, only one small randomized trial has compared conventional dose chemotherapy with high-dose chemotherapy supported by stem cell rescue (in this case ABMT), and this demonstrated improved disease-free survival in the high-dose arm (Humblet et al, 1987). As such, a dose-intensive approach may yield a survival advantage for selected patients and, for these, MVP chemotherapy would represent undertreatment.

However, for older, poor performance status/ED patients, reduction in toxicity with maintenance (ideally with improvement) of the survival fraction is the current goal of trials. The possibility that a 'more gentle' chemotherapy regimen may have inadequate activity in SCLC was demonstrated in a recent trial in which patients with advanced disease were randomized to receive either weekly carboplatin and teniposide or cisplatin, doxorubicin and etoposide alternating with cyclophosphamide, methotrexate, vincristine and lomustine (Joss et al, 1995). The trial was closed before the planned accrual because of a significant survival difference in favour of the more intensive alternating regimen (1 year survival: 30% vs 4%). Toxicity was greater with the more intensive regimen but there was no difference found in patient-related tumour symptoms or general quality-of-life categories. Likewise, that a regimen initially conceived to have low toxicity with acceptable anti-tumour activity may not do so is exemplified by the MRC randomized trial of oral etoposide in comparison to intravenous multidrug chemotherapy (Girling et al, 1996). In this trial, the oral etoposide schedule was associated with greater toxicity and a poorer median survival. However, another MRC randomized trial of a two-drug regimen (EV: etoposide, vincristine) with a four-drug regimen (ECMV: etoposide, cyclophosphamide, methotrexate, vincristine) for poor performance status patients demonstrated how toxicity can be reduced significantly without a survival deficit (Bleehen et al, 1996).

The anti-tumour response achieved with the MVP regimen in this trial was matched by symptom relief and patients overall had an improvement in a range of quality-of-life items (during the initial 12 weeks of treatment), as measured by the EORTC Quality of Life Assessment instrument.

As we have reported previously (Ellis et al, 1995*a*), this MVP regimen has a low cost. Furthermore it is pragmatic and represents a reasonable alternative when diagnostic uncertainties arise and

© Cancer Research Campaign 1998

the tumour cannot be confidently defined as small-cell or non-small-cell.

Moderate-dose MVP chemotherapy has acceptable anti-tumour activity and toxicity in small-cell lung cancer. It offers an appropriate comparator in future randomized trials for poor performance status patients in which minimization of toxicity and enhancement of quality of life are emphasized.

### REFERENCES

Bishop JF, Raghavan D, Stuart Harris R, Morstyn G, Aroney R, Kefford R, Yuen K, Lee J, Gianoutsos P, Olver IN, Zalcberg J, Ball D, Bull C and Fox R (1987) Carboplatin (CBDCA, JM-8) and VP-16-213 in previously untreated patients with small-cell lung cancer. *J Clin Oncol* **5**: 1574–1578

Bleehen NM, Girling DJ, Hopwood P, Lallemand B, Machin RJ and Bailey AJ for the Medical Research Council Lung Cancer Working Party (1996) Randomised trial of four-drug vs less intensive two-drug chemotherapy in the palliative treatment of patient with small-cell lung cancer (SCLC) and poor prognosis. *Br J Cancer* **73**: 406–413

Broder LE, Sridhar KS, Selawry OS, Charyulu KN, Rao RK, Saldana MJ, Donnelly EJ and Raub WA (1994) A randomised clinical trial in bronchogenic small-cell carcinoma evaluating alternating maintenance therapy of vincristine, adriamycin, procarbazine and etoposide (VAPE) with maintenance CCNU and methotrexate (CCM) versus CCM maintenance alone in complete responders following VAPE induction and late intensification. *Am J Clin Oncol* **17**: 527-537

Brugger W, Frommhold H, Pressler K, Mertelsmann R and Kanz L (1995) Use of high-dose etoposide/ifosfamide/carboplatin/epirubicin and peripheral blood progenitor cell transplantation in limited-disease small cell lung cancer. *Semin Oncol* **22**: 3–8

Ellis PA, Smith IE, Hardy JR, Nicolson MC, Talbot DC, Ashley SE and Priest K (1995*a*). Symptom relief with MVP (mitomycin C, vinblastine and cisplatin) chemotherapy in advanced non-small-cell lung cancer. *Br J Cancer* **71**: 366–370

Ellis PA, Talbot DC, Priest K, Jones AL and Smith IE (1995*b*) Dose intensification of carboplatin and etoposide as first-line combination chemotherapy in small cell lung cancer (letter). *Eur J Cancer* **31a**: 1888–1889

Evans WK, Eisenhauer E, Hughes P, Maroun JA, Ayoub J, Shepherd FA and Feld R (1988) VP-16 and carboplatin in previously untreated patients with extensive small cell lung cancer: a study of the National Cancer Institute of Canada Clinical Trials Group. *Br J Cancer* **58**: 464–468

Fetscher S, Brugger W, Englehardt R, Kanz L, Hasse J, Frommhold H, Wenger M, Lange W and Mertelsmann R (1997) Dose-intense therapy with etoposide, ifosfamide, cisplatin, and epirubicin (VIP-E) in 100 consecutive patients with limited- and extensive-disease small-cell lung cancer. *Ann Oncol* **8**: 49–56

Fukuoka M, Furuse K, Saijo N, Nishiwaki Y, Ikegami H, Tamura T, Shimoyama M and Suemasu K (1991) Randomized trial of cyclophosphamide, doxorubicin, and vincristine versus cisplatin and etoposide versus alternation of these regimens in small-cell lung cancer. *J Natl Cancer Inst* **83**: 855–861

Girling D, Thatcher N, Clark PI, Hopwood P, Twiddy S, Stephens RJ, Bailey AJ, Machin D, Bleehen NM, Bolger JJ, Connolly CK, Haselton PS, Macbeth FR, Moghissi K, Saunders MI, White RJ, Alcock S, Burt H, Crossley E, Decker E, English J, Foster G, Hannigan K, Heron D, Hutchinson J, for the Medical Research Council Lung Cancer Working Party (1996) Comparison of oral etoposide and standard intravenous multidrug chemotherapy for small-cell lung cancer: a stopped multicentre randomised trial. *Lancet* **348**: 563–566

Harper PG and Souhami RL (1985) Intensive chemotherapy with autologous bone marrow transplantation in small cell carcinoma of the lung. *Recent Results Cancer Res* **97**: 146–156

Humblet Y, Symann M, Bosly A, Delaunois L, Francis C, Machiels J, Beauduin M, Doyen C, Weynants P, Longueville J and Prignot J (1987) Late intensification chemotherapy with autologous bone marrow transplantation in selected small-cell carcinoma of the lung: a randomized study. *J Clin Oncol* **5**: 1864–1873

Ihde DC, Deisseroth AB, Lichter AS, Bunn PA, Carney DN, Cohen MH, Veach SR, Makuch RW, Johnston-Early A, Abrams RA, Messerschmidt GL, Matthews MJ and Minna JD (1986) Late intensive combined modality therapy followed by autologous bone marrow infusion in extensive-stage small-cell lung cancer. *J Clin Oncol* **4**: 1443–1454

Inada T (1988) In vitro chemosensitivity tests of human lung small cell carcinomas – with reference to combination cancer chemotherapy. *Nippon Geka Gakkai Zasshi* **89**: 256–264

Jones AL, Holborn J, Ashley S and Smith IE (1991). Effective new low toxicity chemotherapy with carboplatin, vinblastine and methotrexate for small cell lung cancer: a randomised trial against doxorubicin, cyclophosphamide and etoposide. *Eur J Cancer* **27**: 866–870

Jones AL, Holborn J, Ashley S and Smith IE (1993) CVM versus ACE in the treatment of small cell lung cancer. *Oncology* **50**: 10–15

Joss RA, Alberto P, Hurny C, Bacchi M, Leyvraz S, Thurlimann B, Cerny T, Martinelli G, Stahel R and Ludwig C (1995) Quality versus quantity of life in the treatment of patients with advanced small-cell lung cancer? A randomized phase III comparison of weekly carboplatin and teniposide versus cisplatin, adriamycin, etoposide alternating with cyclophosphamide, methotrexate, vincristine and lomustine. Swiss Group for Clinical Cancer Research (SAKK). *Ann Oncol* **6**: 41–48

Kaplan EL and Meier P (1958) Nonparametric estimation from incomplete observations. *J Am Stat Assoc* **53**: 457–481

Klasa RJ, Murray N and Coldman AJ (1991) Dose-intensity meta-analysis of chemotherapy regimens in small-cell carcinoma of the lung. *J Clin Oncol* **9**: 499–508

Leyvraz S, Ketterer N, Perey L, Bauer J, Vuichard P, Grob JP, Schneider P, von Fliedner V, Lejeune F and Bachmann F (1995) Intensification of chemotherapy for the treatment of solid tumours: feasibility of a 3-fold increase in dose intensity with peripheral blood progenitor cells and granulocyte colony-stimulating factor. *Br J Cancer* **72**: 178–182

McHale MS and Einhorn LH (1986) Phase II study of mitomycin in small cell lung carcinoma. *Cancer Treat Rep* **70**: 675

Miller AB, Hoogstraten B, Staquet M and Winkler, A (1981) Reporting results of cancer treatment. *Cancer* **47**: 207–214

Murray N, Shah A, Wilson K, Goldie J, Voss N, Fryer C, Klimo P, Coy P, Hadzic E, Gudauskas G and Fowler R (1985) Cyclic alternating chemotherapy for small cell carcinoma of the lung. *Cancer Treat Rep* **69**: 1241–1242

Roth BJ, Johnson DH, Einhorn LH, Schacter LP, Cherng NC, Cohen HJ, Crawford J, Randolph JA, Goodlow JL, Broun GO, Omura GA and Greco FA (1992) Randomized study of cyclophosphamide, doxorubicin, and vincristine versus etoposide and cisplatin versus alternation of these two regimens in extensive small-cell lung cancer: a phase III trial of the Southeastern Cancer Study Group. *J Clin Oncol* **10**: 282–291

Smith IE (1992) Carboplatin in small cell lung cancer. *Semin Oncol* **19**: 24–27

Smith IE, Evans BD, Harland SJ, Robinson BA, Yarnold JR, Glees JG and Ford HT (1985) High-dose cyclophosphamide with autologous bone marrow rescue after conventional chemotherapy in the treatment of small cell lung carcinoma. *Cancer Chemother Pharmacol* **14**: 120–124

Smith IE, Evans BD, Gore ME, Repetto L, Yarnold JR and Ford HT (1987) Carboplatin (Paraplatin; JM8) and Etoposide (VP-16) as first-line combination therapy for small-cell lung cancer. *J Clin Oncol* **5**: 185–189

Smith IE, Perren TJ, Ashley SA, Woodiwiss J, Forgeson GV, Yarnold JR and Ford HT (1990) Carboplatin, etoposide, and ifosfamide as intensive chemotherapy for small-cell lung cancer. *J Clin Oncol* **8**: 899–905

Woll PJ, Hodgetts J, Lomax L, Bildet F, Cour Chabernaud V and Thatcher N (1995) Can cytotoxic dose-intensity be increased by using granulocyte colony-stimulating factor? A randomized controlled trial of lenograstim in small-cell lung cancer. *J Clin Oncol* **13**: 652–659

© Cancer Research Campaign 1998